# Strategy of Salt Tolerance and Interactive Impact of *Azotobacter chroococcum* and/or *Alcaligenes faecalis* Inoculation on Canola (*Brassica napus* L.) Plants Grown in Saline Soil

**DOI:** 10.3390/plants10010110

**Published:** 2021-01-07

**Authors:** Arafat Abdel Hamed Abdel Latef, Amal M. Omer, Ali A. Badawy, Mahmoud S. Osman, Marwa M. Ragaey

**Affiliations:** 1 Department of Biology, Turabah University College, Turabah Branch, Taif University, P.O. Box 11099, Taif 21944, Saudi Arabia; 2 Desert Research Center, Department of Soil Fertility and Microbiology, El-Matareya 11753, Cairo, Egypt; amal_omaram@yahoo.com; 3 Botany and Microbiology Department, Faculty of Science, Al-Azhar University, Nasr City 11884, Cairo, Egypt; 4 Botany and Microbiology Department, Faculty of Science, New Valley University, Al-Kharja 72511, New Valley, Egypt; marwa_ragaey@sci.nvu.edu.eg

**Keywords:** plant growth-promoting rhizobacteria, salinity, canola, osmolytes, antioxidant activity

## Abstract

A pot experiment was designed and performed in a completely randomized block design (CRBD) to determine the main effect of two plant growth-promoting rhizobacteria (PGPR) and their co-inoculation on growth criteria and physio-biochemical attributes of canola plants (*Brassica napus* L.) plant grown in saline soil. The results showed that inoculation with two PGPR (*Azotobacter chroococcum* and/or *Alcaligenes faecalis*) energized the growth parameters and photosynthetic pigments of stressed plants. Moreover, soluble sugars’ and proteins’ contents were boosted due to the treatments mentioned above. Proline, malondialdehyde (MDA), and hydrogen peroxide (H_2_O_2_) contents were markedly declined. At the same time, antioxidant enzymes, viz. superoxide dismutase (SOD), ascorbate peroxidase (APX), and peroxidase (POD), were augmented due to the inoculation with *Azotobacter chroococcum* and/or *Alcaligenes faecalis*. Regarding minerals’ uptake, there was a decline in sodium (Na) and an increase in nitrogen (N), potassium (K), calcium (Ca), and magnesium (Mg) uptake due to the application of either individual or co-inoculation with the mentioned bacterial isolates. This study showed that co-inoculation with *Azotobacter chroococcum* and *Alcaligenes faecalis* was the most effective treatment and could be considered a premium tool used in facing environmental problems, especially saline soils.

## 1. Introduction

Soil salinity directly affects crops. It is one of the most destructive abiotic stresses due to its disastrous effect on agricultural areas and reducing crops’ quality and productivity [1,2,3,4]. Moreover, there is also an abnormal increase in saline soils. This increase is due to several reasons, including some unfavorable agricultural practices, irrigation with saline water, and high surface evaporation rate [5].

Salinity of the soil limits crop plants’ productivity depending on the crop plants’ sensitivity to salts concentrations. Salt-stressed soils reduce plant growth [6,7]. They can also interfere with nitrogen (N) nutrition in the plant in direct or indirect ways, usually at the inorganic nitrogen compounds’ assimilation pathway. Besides, where high concentrations of salts are present in soils, the capacity for NO_3_ leaching in soil may boost because the plants’ efficiency in absorbing or utilizing the applied N from the soil is reduced under salinity stress [8]. One of the most severe problems that depend on salinity is the accumulation of reactive oxygen species (ROS) that leads to oxidative stress causing oxidative damage of proteins, pigments, and DNA of salt-stressed plants [7,9,10,11]. The higher concentration of salt in soil affects the plant’s potency and efficiency to absorb water and essential nutrients by roots. High sodium concentration inside the plant cell leads to many disturbances that lead to a decrease in plant growth [12,13]. Excess salt concentration decreased photosynthetic pigments in plant leaves, leaf area, and photosynthetic efficiency [3,14]. Furthermore, salinity stress caused oxidative stress due to the accumulation of hydrogen peroxide (H_2_O_2_), which induces cell shrinkage, DNA fragmentation, and induce malondialdehyde (MDA) accumulation, which is represented as an indicator for lipid peroxidation [15].

Plant growth-promoting bacteria (PGPB) play a direct or indirect useful role in enhancing plant growth, yield, and nutrient uptake through various action mechanisms [4,16]. These bacterial strains directly regulate plant physiology by promoting the nutrient uptake through phytohormone production (e.g., auxin, gibberellins, and cytokinin), increasing nitrogen and mineral availability in the soil and/or producing siderophores [17]. The PGPB containing 1-aminocyclopropane 1-carboxylate (ACC) deaminase are located in various soils and offer a promising approach for improving plant growth, particularly under stressed environmental conditions. Plants inoculated with ACC-deaminase containing PGBR showed a decrease in stimulated ethylene due to the diminishing impact of salt stress on ethylene. Plants with a lower level of ethylene showed more excellent resistance to abiotic stress [18]. Therefore, it could be mentioned that plants treated with ACC-deaminase containing PGPB help different plants to face different types of abiotic stresses [19,20].

*Azotobacter* genus is characterized as a free-living, aerobic, nitrogen-fixer, heterotrophic, Gram-negative bacteria in the class γ-proteobacteria. The first described species in *Azotobacter* genus was *A. chroococcum* [21]. Inoculation with *A. chroococcum* improves crop resistance to salinity through increasing plant content of soluble sugars, soluble proteins, and proline in shoots and roots. Moreover, it stimulates plant growth by increasing the dry weights of root and shoot [22,23,24]. *Alcaligenes faecalis* was isolated first in 1896, an *Alcaligenaceae* family member. This species is Gram-negative rods that are aerobically motile, flagellated, slightly curved non-spore-forming, slowly growing, and capsule-forming bacteria [25]. *Alcaligenes faecalis* is considered PGPB due to its ability to produce indole acetic acid (IAA), ACC-deaminase, and phosphate solubilization and fix atmospheric nitrogen [26]. Also, [27] showed that *Alcaligenes* sp. could be used as a biofertilizer to enhance the growth and yield of different plants under typical and different stress types. Inoculation with *Alcaligenes faecalis* containing ACC-deaminase ameliorates the salinity stress effect on growth, biochemistry, and yield of plants [28]. The most prevalent reason for the impact of *Alcaligenes faecalis* on plants is based on the production of phytohormones that alter plant morphology and metabolism, leading to improved water and mineral absorption [26].

Canola (*Brassica napus* L.), also known as oilseed rape, is one of the most important oilseed crops globally and was ranked globally as third in the term of oilseed crop production following soybean and palm oil [29,30]. At the same time, it ranks first among field oil crops that tolerate stressed conditions [31]. Canola seeds contain 40–42% oil, 60% oleic acid, 8.8% linoleic acid, and 25% protein [32,33,34]. Cultivation of canola in Egypt can introduce an opportunity to beat several deficiencies in edible oil production. Additionally, canola could be successfully cultivated in newly reclaimed land out of the old Nile Valley areas to avoid competition with other crops inhabiting the old cultivated lands [35,36].

Because of damage caused by salinity to crops and due to increases in saline land area, overcoming this problem in Egypt became one of the most critical challenges; therefore, it was necessary to use one of the appropriate approaches to meet this challenge. So, the use of plant growth-promoting rhizobacteria (PGPR) was considered as an alternative tool to alleviate salinity stress of essential oil crops like canola. In this line, we examined the possible role of PGPR strains *Azotobacter chroococcum* and *Alcaligenes faecalis* (individually or in co-inoculation) in enhancing salinity tolerance in canola plants by evaluating their impact on growth attributes, the contents of photosynthetic pigments, osmolytes, oxidative stress, and minerals as well as the antioxidants’ enzyme activities.

## 2. Results

### 2.1. Microbiological Characteristics in the Canola Rhizosphere

Results in Table 1 show the positive effect of bacterial inoculation on both the microbial community’s abundance and activity in the canola rhizosphere. Under salinity-stress regimes, total microbial count in canola rhizosphere was enhanced in response to inoculation with *Azotobacter chroococcum* and *Alcaligenes faecalis* and their mixture by 65.5%, 110.3%, and 113.7%, respectively. Moreover, nitrogen fixer count was increased in the treatments, as mentioned earlier, by 3.6%, 3.9%, and 3.9%, respectively. Dehydrogenase activity in the rhizosphere of *Azotobacter chroococcum-* and *Alcaligenes faecalis*-inoculated plants was increased by 63.3% and 116.6%, while co-inoculation recorded an increase in dehydrogenase activity by 112%.

### 2.2. PGPR Enhance Canola Plant Growth under Salinity Stress

**Figure 1:**
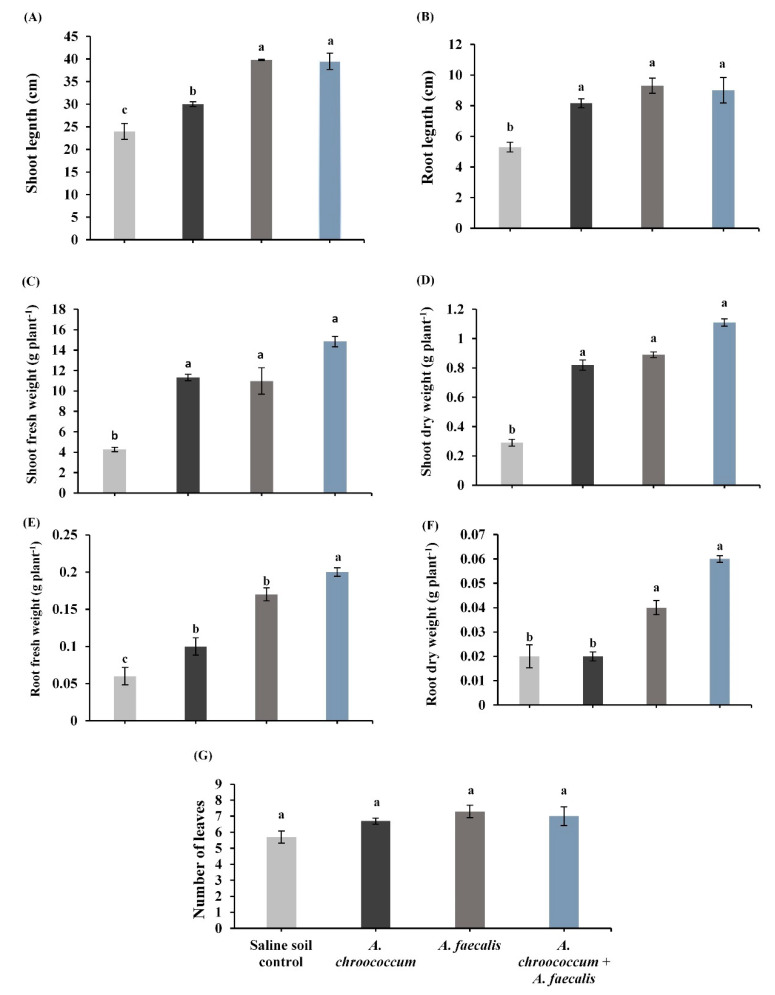
(**A**) shoot length; (**B**) root length; (**C**) shoot fresh weight; (**D**) shoot dry weight; (**E**) root fresh weight; (**F**) root dry weight; and (**G**) number of leaves of canola plant inoculated with *Azotobacter chroococcum, Alcaligenes faecalis,* and their co-inoculation under salinity-stress conditions. Bars show means of three independent replications (n = 3) ± standard error. Means with the same letter are not significantly different at *p* < 0.05.

The results in Figure 1 show that the rhizobacterial inoculation with *A. chroococcum*, *A. faecalis,* and their co-inoculation enhanced the different canola growth parameters such as lengths of shoot and root, fresh and dry weights of shoot, fresh and dry weights of root, and number of leaves. Significant increases recorded by the individual inoculation with *A. chroococcum* were observed in shoot length by 25%, root length by 54%, shoot fresh weight by 165.7%, shoot dry weight by 182.8%, and root fresh weight by 66.70% as compared to un-inoculated canola plants grown in saline soil control. Moreover, the inoculation with *A. faecalis* recorded significant increases in shoot length, root length, shoot fresh weight, shoot dry weight, and root fresh weight by 65.8%, 75.5%, 157.5%, 93.1%, and 183.3%, respectively. The co-inoculation between the two mentioned strains showed significant increases reached to 64.6% in shoot length, 69.8% in root length, 248.4% in shoot fresh weight, 282.8% in shoot dry weight, 233.3% in root fresh weight, and 200% in root dry weight of salinity-stressed canola plants in comparison with saline soil control. Results in Figure 1 show that bacterial inoculation with *A. chroococcum*, *A. faecalis,* and their mixture insignificantly enhanced the number of leaves in canola plants under salinity-stress conditions.

### 2.3. PGPR Protect Photosynthetic Pigments in Leaves of Canola Plant under Salinity Stress

**Figure 2:**
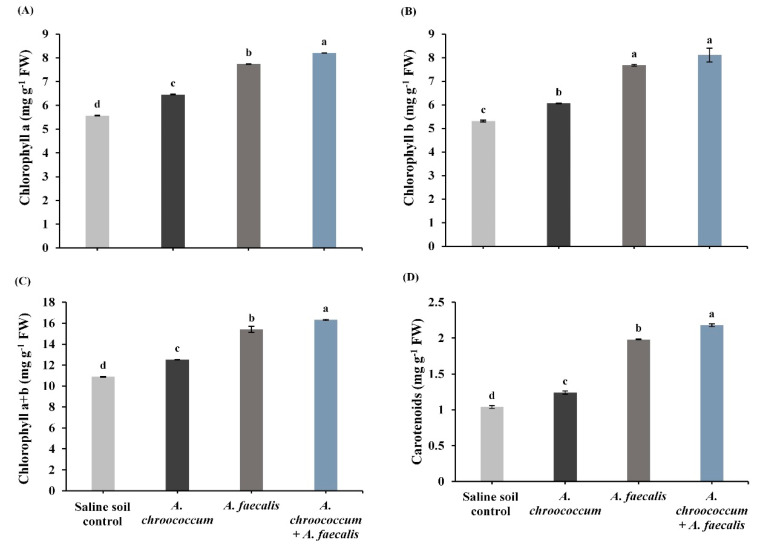
(**A**) Chlorophyll a; (**B**) chlorophyll b; (**C**) chlorophyll a + b; and (**D**) carotenoids’ content in canola plant fresh leaves inoculated with *Azotobacter chroococcum, Alcaligenes faecalis,* and their co-inoculation under salinity-stress conditions. Bars show means of three independent replications (n = 3) ± standard error. Means with the same letter are not significantly different at *p* < 0.05. FW: fresh weight.

Both bacterial strains, *A. chroococcum* and *A. faecalis,* markedly accumulated fresh leaves’ contents of chlorophyll a (by 16% and 39%), chlorophyll b (by 14.1% and 44.6%), total chlorophyll (by 15.1% and 41.6%), and carotenoids (by 19.2% and 90.4%), respectively, when compared to saline soil control (Figure 2). The results exhibited that the highest significant increases in chlorophyll a (by 47.4%), chlorophyll b (by 52.7%), total chlorophyll (by 50%), and carotenoids (by 109.6%) were recorded in response to the co-inoculation with the two strains when compared to saline soil control.

### 2.4. PGPR Regulate Osmolytes’ Contents in Salinity-Stressed Canola Plants

Soluble sugars’ content of stressed canola plants was insignificantly enhanced in response to the individual inoculation with *A. chroococcum* or *A. faecalis* isolate, while soluble sugars’ content was significantly augmented, by 94% in the case of the two isolates’ interaction when compared with saline soil control (Table 2). The inoculation with PGPR changed the soluble proteins’ content of canola plants that were cultivated in saline soil. The inoculation of stressed canola plants with *A. chroococcum*, *A. faecalis,* or their interaction respectively recorded significant increases by 45.5%, 50.9%, and 55% of soluble proteins content (Table 2). Proline content was insignificantly decreased due to the treatment with *Azotobacter chroococcum* strain (Table 2). The decreases of proline content in salinity-stressed canola plants were recorded by 7.8% and 10.9% when the plants were inoculated with *Alcaligenes faecalis* and the interaction (Table 2).

### 2.5. PGPR Lessen MDA and H_2_O_2_ Contents in Leaves of Salinity-Stressed Canola Plants

**Figure 3:**
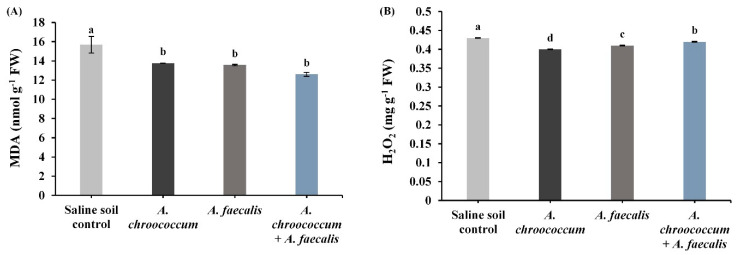
(**A**) Malondialdehyde (MDA) content and (**B**) hydrogen peroxide (H_2_O_2_) content in canola plant fresh leaves inoculated with *Azotobacter chroococcum*, *Alcaligenes faecalis*, and their co-inoculation under salinity-stress conditions. Bars show means of three independent replications (n = 3) ± standard error. Means with the same letter are not significantly different at *p* < 0.05. FW: fresh weight.

Comparing with the non-inoculated plants, MDA and H_2_O_2_ contents of salinized canola plants were inhibited due to the application of *A. chroococcum* about 12% and 7%, *A. faecalis* by 13.5% and 4.7%, and their interaction by 19.6% and 2.3%, respectively (Figure 3).

### 2.6. PGPR Stimulate Antioxidant Enzymes under Salinity-Stress Conditions

**Figure 4:**
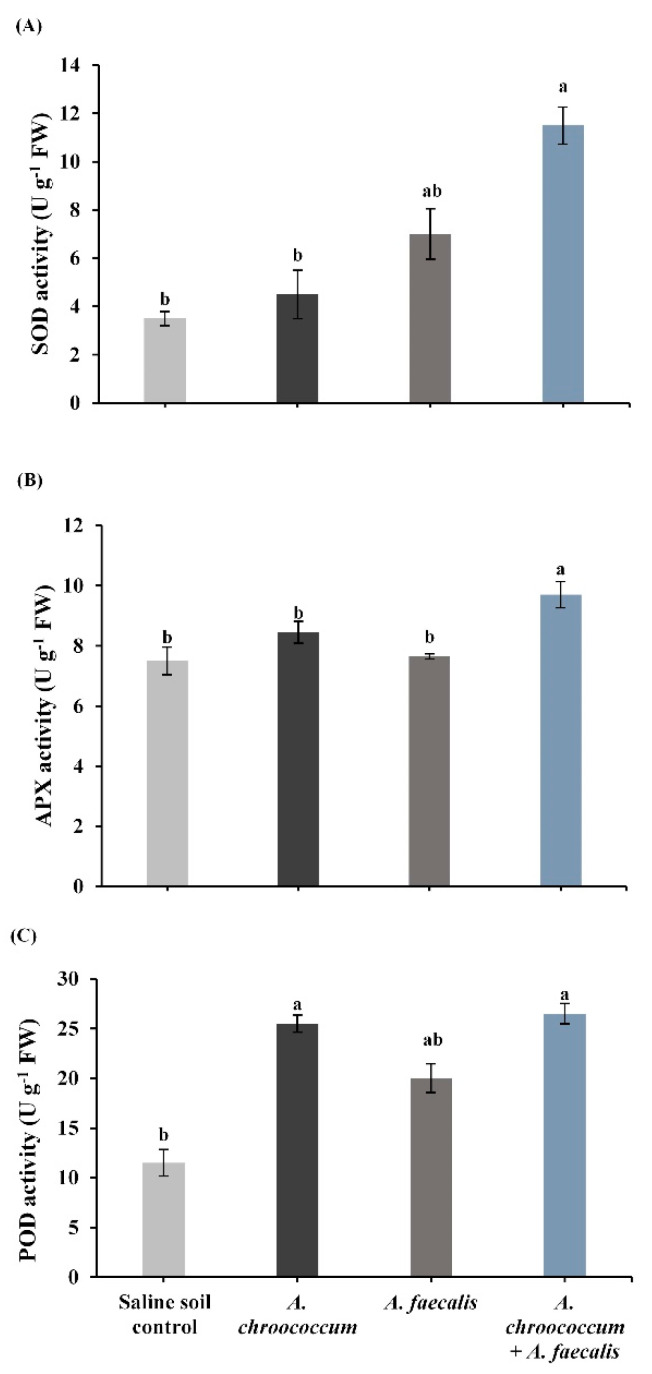
(**A**) Superoxide dismutase (SOD), (**B**) ascorbate-peroxidase (APX), and (**C**) peroxidase (POD) activity in canola plant fresh leaves inoculated with *Azotobacter chroococcum, Alcaligenes faecalis,* and their co-inoculation under salinity-stress conditions. Bars show means of three independent replications (n = 3) ± standard error. Means with the same letter are not significantly different at *p* < 0.05. FW: fresh weight.

The inoculation with *Azotobacter chroococcum* insignificantly stimulated the levels of SOD and APX of salinity-stressed canola plants, while it significantly stimulated POD by 121.7% (Figure 4). Regarding the inoculation with *Alcaligenes faecalis*, the activities of SOD, APX, and POD were insignificantly enhanced. In the interaction treatment with the two bacterial strains, there was a significant enhancement in SOD by 228.6%, APX by 29.3%, and POD by 130.4% of canola plants grown in saline soil in comparison with un-inoculated plants (Figure 4).

### 2.7. PGPR Regulate Mineral Uptake in Salinity-Stressed Canola Plants

Under salinity-stress conditions, canola plants inoculated with *Azotobacter chroococcum, Alcaligenes faecalis,* and their co-inoculation significantly decreased sodium (Na) content by 50.31%, 37.62%, and 57%, respectively (Table 3). However, the content of potassium (K) was increased dramatically due to the treatments as mentioned above by 51.98%, 77.98%, and 41.47%, respectively (Table 3), as compared to saline soil control. The contents of nitrogen (N) and calcium (Ca) were significantly increased in response to inoculation with *Azotobacter chroococcum* by 57.46% and 11.76%, respectively, while the content of magnesium (Mg) was decreased by 21.19% (Table 3). Moreover, inoculation with microbial strain *Alcaligenes faecalis* significantly increased contents of N, Ca, and Mg by 46.84%, 33.82%, and 80.50%, respectively (Table 3). Co-inoculation with *Azotobacter chroococcum* and *Alcaligenes faecalis* enhanced the contents of N, Ca, and Mg by 79.28%, 175%, and 55.08% versus saline soil control (Table 3).

## 3. Discussion

Salinization of water and soil plays a crucial role in limiting crops’ growth and productivity [3,4,37]. Soil salinity is a vast problem that spreads in most areas over the world, so it is imperative to found solutions for plants to have the ability to grow in these salinized areas [38]. In recent years, light has been shed on the use of natural sources and microorganisms (bacteria, fungi, algae, plant extracts, etc.) to cope with salt stress and mitigate its harmful effects on plant life [4,7,39,40,41,42]. PGPB have the colossal ability to lessen salt stress and improve plant development, playing a critical role in food security by boosting the productivity of crops. Use of PGPB under salinity stress enhances plant growth in several ways, including ACC deaminase activity, synthesis of plant hormones as IAA, gibberellic acid (GA), abscisic acid (ABA), cytokinin, and exopolysaccharides [43]. PGPR stimulate plant growth directly by enhancing the uptake of nutrients through phytohormone production (e.g., auxin, gibberellins, and cytokinin) or by lowering plant ethylene levels enzymatically [17]. It has been suggested that the production of auxins by root-associated microbes is one of the most important mechanisms through which microbes regulate plant growth. Also, specific beneficial endophytes can produce auxin and/or display ACC deaminase activity that can aid host plant growth in dangerous areas [44].

Soil salinity as a vital stress factor harms the microbial process, diminishing bacterial diversity and controlling microbial wealth, composition, and functions [28]. Plants’ inoculation can mitigate this negative impact of salinity with our tested PGPR *Azotobacter chroococcum*, *Alcaligenes faecalis,* and co-inoculation. Bacterial treatment of canola plants with plant growth-promoting rhizobacteria had a remarkable stimulation effect on the rhizosphere’s microbial population [45]. Dehydrogenase activity by indigenous microorganisms in soil can serve as a valuable marker of microbial activity, which indicates the relative effectiveness of microbes with plant rhizosphere in soils [46]. Our results are in harmony with results [47] that stated that the combined inoculation with *Azospirillum* sp. and *Bacillus* sp. increased the dehydrogenase at all growth plant stages. Plant growth-promoting rhizobacteria can enhance the tolerance of plants to various abiotic stresses, including salinity.

Our study demonstrated enhancements in salinity-stressed canola plants’ growth parameters in response to inoculation with *Azotobacter chroococcum* and *Alcaligenes faecalis*. Similar improvements in plant growth due to inoculation with halotolerant plant growth-promoting bacterium *Alcaligenes faecalis* were evidenced in the study of [48], which stated that vegetative growth characteristics of salinity-stressed rice and wheat plants, respectively, were increased. These results documented that PGPR inoculation appeared to reinforce canola’s growth by relieving the suppression caused by salinity stress [49]. The utilization of PGPR was recommended to boost the growth of different salinity-stressed crops [50,51,52,53]. Ref. [54,55] linked the augmentation of plant growth with the ability of PGPR to produce some plant growth regulators, solubilize phosphate, and fix nitrogen. These features are found in the selected isolates and generally increase a plant’s ability to absorb nutrients from the soil and improve its growth, especially under salinity-stress conditions.

Photosynthetic pigments are a fundamental physiological trait directly associated with photosynthesis ability under abiotic stresses. Our results observed increases in chlorophylls and carotenoids in canola plants cultivated in saline soil. These increases were due to the soil supplementation with the tested PGPR. Similar results recorded enhancements in the photosynthetic pigments in PGPR-inoculated plants under different saline conditions [56,57,58]. The augmentation in photosynthetic pigments in PGPR-inoculated plants suggests the potency of bacterial inoculation to nullify the harmful impacts of salinity stress by improving the activities of electron transporters associated with photosynthesis [59] as well as the biosynthesis of proteins and enzymes that related to pigment stabilization [60].

In the present study, the inoculation with plant growth-promoting rhizobacterial strains *Azotobacter chroococcum* and *Alcaligenes faecalis,* especially the co-inoculation between them, led to enhancements in soluble sugars’ content in canola plants cultivated in saline soil. These enhancements were evidenced in several studies [61,62,63,64,65]. Recently, the inoculation with *A. chroococcum* exhibited increases in sugar contents in maize plants cultivated in salt-affected soil [4]. They documented that sugar content rising is considered as a vital osmolyte that maintains the plant against salinity stress. The current study clarified that soluble proteins’ content in salinity-stressed canola plants was increased due to the inoculation with PGPR. Various studies on crop plants have well documented the positive impacts of rhizobacterial inoculation on increasing the soluble protein content [4,63,64,65]. A possible strategy behind this increase could be that bacterial inoculation might inhibit the activity of protein-hydrolyzing enzymes in addition to the ability of bacteria in promoting the efficiency of proline in protecting soluble proteins and, thus, increasing their amounts under the salt-stress conditions [63,65,66].

The plant faces environmental stressors by accumulating some osmolytes like proline, which acts as a solute for osmoregulation [67]. However, the accumulation of proline in plants has been documented as an environmental stress indicator [68]. The proline level in the present study was inhibited in salinity-stressed canola plants that were inoculated with the tested PGPR containing ACC-deaminase. This result is in harmony with the findings of [69,70]. The current results may imply that PGPR alleviated the severity of salinity stress on the plant and, thus, the proline content (a marker of stress) in canola shoots also lessened.

Our study showed a reduction in the contents of MDA and H_2_O_2_ in salinized canola plants that were inoculated with PGP rhizobacterial isolates. Our findings on the efficacy of PGPR in decreasing the contents of MDA and H_2_O_2_ in plants cultivated in conditions of salinity stress are in harmony with the studies of [52,70,71]. Thus, PGPR could prevent canola plants from oxidative destruction caused by salinity stress.

To mitigate the oxidative stress induced by salinity stress, the plants developed a group of physiological and biochemical strategies made of various enzymes that can scavenge the ROS species. Antioxidant enzymes act in a network to achieve the detoxification of ROS species [10,72,73]. In our study, we noticed different increases in SOD, APX, and POD activities in the inoculated canola plants with the mentioned PGPR under saline conditions. Our findings comply with the reports of [74] on mung bean.

In this study, PGPR’s positive role appeared in removing the harmful effect of salinity stress by limiting the uptake of Na and increasing the uptake of essential minerals such as N, K, Ca, and Mg. This positive role may be attributed to the finding that PGPR facilitate the entity of essential elements in the soil to be easily absorbed by the plant [13,75] or due to roots’ exudates initiated by PGPR, increasing the availability of some micronutrients [57,76]. Moreover, increasing nitrogen content in canola shoots is attributed to the ability of PGPR in increasing nitrogen and mineral availability in the soil [17].

## 4. Materials and Methods

### 4.1. Isolation, Identification, and Description of PGPR (Salt-Tolerant Bacteria)

Two halophilic bacterial strains were used for alleviating the salt stress in canola plants. The first halophilic strain is rhizospheric bacteria isolated, purified on Ashby’s media as selective media [77] from the rhizosphere of wheat plant cultivated in saline soil at Sahl El-Tina, Sinai, Egypt (Electrical conductivity (EC 6000–7000 ppm), and identified to its molecular level as *Azotobacter chroococcum* strain NBRC using partial 16S rRNA gene sequence technique according to [78] in Sigma Scientific Services Co. (Giza, Egypt). The second strain is rhizospheric bacteria isolated, purified on King’s media as selective media [79] from the barley plant’s rhizosphere cultivated in saline soil at Ras Sudr, Sinai, Egypt (EC 5000–6000 ppm), and identified to molecular level as *Alcaligenes faecalis* strain NBRC 13111. Two strains were assigned in Gene Bank NCBI with accession number as NR 113606.1 and NR114167, respectively.

### 4.2. Pot Experiment

A pot experiment was conducted in the greenhouse of the microbiological unit of Desert Research Center, Cairo, Egypt. Canola (*Brassica napus* L. cv. Pactol) was provided by Agricultural Research Center (ARC), Giza, Egypt. Microbial inoculants of *Azotobacter chroococcum, Alcaligenes faecalis,* and a mixture of them were used for treating canola plants. The experimental design was performed in a complete randomized block design (CRBD) with three replications. Climate conditions were: average day/night temperature cycle of 23/12, light 10/14 h, and air humidity between 39% and 58%. Ten Seeds of canola were planted into 10-kg pots containing saline soil collected from Sahl El-Tina, Sinai, Egypt. The physical and chemical characteristics of the soil were soil depth 0–15 cm^−1^, total sand 30%, silt 10.2%, clay 59.8%, texture clay, EC 11.5 mmhos cm^−1^, pH 7.6, HCO_3_- 18.5 mg g^−1^, Cl^-^ 51.6 mg g^−1^, SO_4_^−^ 9.8 mg g^−1^, Ca^2+^ 21.1 mg g^−1^, Mg^2+^ 15.1 mg g^−1^, Na^+^ 56.2 mg g^−1^, and K^+^ 0.64 mg g^−1^. For bacterial treatments, seeds were coated with bacterial inoculum using carboxymethyl cellulose (CMC) solution (1%) in the ratio of 1 kg seeds/250 mL of inoculum (10^6^ CFU/mL) mixed with 50 g^−1^ of CMC before application to get a thin, uniform coating of bacterial inoculum on seeds. Inoculated seeds were dried in shade before sowing [80]. Untreated control seeds were maintained. After seed germination, plants were thinned1to five plants per pot then each pot was inoculated with 10 mL of microbial inoculum (10^6^ CFU/mL) of an individual strain and mixture of them. Pots were arranged as follows: (1) saline soil control, (2) saline soil control + *Azotobacter chroococcum*, (3) saline soil control + *Alcaligenes faecalis*, and (4) saline soil control + co-inoculation with *Azotobacter chroococcum* and *Alcaligenes faecalis*. Pots were irrigated two times weekly. After 66 days of planting, the plants were harvested to determine lengths of shoots and roots, fresh and dry weights of shoots and roots, and biochemical parameters.

### 4.3. Microbiological Analysis of Canola Rhizosphere

Total microbial count and populations of *Azotobacter chroococcum* and *Alcaligenes faecalis* in the rhizosphere samples were estimated using yeast extract agar medium [81], Ashby’s [77], and King’s media [79]. Soil dehydrogenase activity (μg TPF/g dry soil/24 h) was analyzed by the reduction of 2,3,5-triphenyl tetrazolium chloride (TTC) to triphenyl formazan (TPF) as described by [82].

### 4.4. Determination of Photosynthetic Pigments

Chlorophyll content in fresh leaves of canola plants was estimated according to methods described by [83]. In this method, 100 mL of acetone (80%) were used for pigments’ extraction from fresh leaves (1 g). Then, the extract was filtered and the green color was measured at 470, 649, and 665 nm using spectrophotometer. Photosynthetic pigments calculated according to the following equations: Chl a (mg g^−1^ FW) = 11.63(A665) − 2.39(A649), Chl b (mg g^−1^ FW) = 20.11(A649) − 5.18(A665); Chl a + b (mg g^−1^ FW) = 6.45 (A665) +1 7.72(A649); carotenoids’ contents (mg g^−1^ FW) = {(1000 ×A470) − (1.82 × Chl a) − (85.02 × Chl b)}/198, according to [84].

### 4.5. Determination of Osmolyte Contents

Soluble sugars’ content of dried canola plants’ shoot was estimated according to [85]. One g from the dried sample was placed with 5 mL of 2% phenol and 10 mL of 30% trichloroacetic acid for extraction. Two mL of the filtered extract were mixed with 4 mL of anthrone reagent (2 g anthrone/L of 95% sulfuric acid). At 620 nm we measured the developed blue-green color.

Soluble proteins’ content was estimated according to methods of [86] in the dried shoot of canola plants. Sample (0.1 g) was extracted in 5 mL of 2% phenol and 10 mL of distilled water. One mL of extract was added to 5 mL of alkaline reagent (50 mL from 2% Na_2_CO_3_ prepared in 0.1 N NaOH and 1 mL from 0.5% CuSO_4_.5H_2_O prepared in 1% sodium potassium tartrate) and mixed thoroughly. Then, 0.5 mL of folin reagent (diluted 1:3 *v*/*v*) was added. The developed color after 30 min was measured at 750 nm.

The described method of [87] was used for determination of proline contents. In such method, a half gram of the dried shoot of canola plants was homogenized in 10 mL (3%) sulfosalicylic acid. The homogenate was filtered and 2 mL of it were reacted with 2 mL of acid ninhydrin (warm 1.25 g ninhydrin in 30 mL glacial acetic acid and 20 mL 6M phosphoric acid) and 2 mL of glacial acetic acid for one hour in a boiling water bath. Then, the reaction was placed in an ice bath. Four mL of toluene was added to the mixture 4. Then, we read the absorbance at 520 nm.

### 4.6. Estimation of Malondialdehyde Content

MDA content in canola fresh leaves was estimated according to the described method of [88]. In this method, fresh leaf samples (0.5 g) were extracted with 5% trichloroacetic acid and centrifugated at 4000× *g* for 10 min. Then, 2 mL of the extract were mixed with 2 mL of 0.6% Thiobarbituric acid (TBA) solution. Then, the mixture was placed in a water bath for 10 min. After cooling, the absorbance of the devolved color was at 532, 600, and 450 nm subsequently. MDA content was calculated according to the following equation: 6.45 × (A532 − A600) − 0.56 × A450.

### 4.7. Determination of Hydrogen Peroxide (H_2_O_2_) Content

Estimation of hydrogen peroxide content in the leaves of canola plants was according to methods described by [89], in which fresh samples (0.05 g) were extracted with 4 mL cold acetone. An aliquot (3 mL) of the extracted solution was mixed with 1 mL of 0.1% titanium dioxide in 20% (*v*:*v*) H_2_SO_4_ and the mixture was then centrifuged at 6000 rpm for 15 min. The intensity of the yellow color of the supernatant was measured at 415 nm.

### 4.8. Extraction and Assay of Antioxidant Enzymes

For the extraction of antioxidant enzymes, terminal buds with first true leaves of canola plants were used, according to methods described by [89], for the extraction of POD and SOD. A method described in [4] was used to extract ascorbate peroxidase (APX). 

The activity of SOD was estimated according to methods described by [90]. The solution (10 mL) consisted of 3.6 mL of distilled water, 0.1 mL of enzyme, 5.5 mL of 50 mM phosphate buffer (pH 7.8), and 0.8 mL of 3 mM pyrogallol (dissolved in 10 mM HCl). The rate of pyrogallol reduction was measured at 325 nm with UV-spectrophotometer.

The described method of [91] was followed to estimate APX activity, in which 0.5 mM AsA, 0.8 mL of potassium phosphate buffer (50 mM, pH 7), 0.1 mM H_2_O_2_, and 0.2 mL enzyme extract were mixed. The changes in absorbance were read at 290 nm.

The activity of POD was estimated according to methods described by [92]: 5.8 mL of 50 mM phosphate buffer (pH 7.0), 0.2 mL of the enzyme extract, and 2 mL of 20 mM H_2_O_2_ after addition of 2 mL of 20 mM pyrogallol. The rate of increase in absorbance as pyrogallol was determined spectro-photometrically by UV-visible spectro-photometer within 60 s at 470 nm.

### 4.9. Determination of Mineral Contents

Dry shoot samples (0.1 g) were acid digested with 80% perchloric acid (HCLO_4_) and sulfuric acid (H_2_SO_4_) 1:5 solution for 12 h. A method described by [93] was used to determine Na, K, Ca, and Mg in the digested sample. Nitrogen content was determined in digested sample according to a modified micro-Kjeldahl method [94].

### 4.10. Statistical Analysis

Data were statistically analyzed by analysis of variance (ANOVA), to determine a significant difference between different treatments using CoStat (CoHort software, Monterey, CA, USA). Least significant difference (LSD) at *p* ≤ 0.05 was used to indicate a significant difference among treatments. Results were shown as mean ± standard error (SE) of three independent replications for each treatment (n = 3).

## 5. Conclusions

From the outcome of the obtained results, it seems likely to conclude that using of *Azotobacter chroococcum* and *Alcaligenes faecalis* brought about enhancements in different growth indices of canola plants grown in saline soil. The co-inoculation with both bacterial isolates brought about significant improvements in most morphology parameters, photosynthetic pigments and carotenoids, soluble sugars, and soluble protein contents. Also, proline, malondialdehyde, and hydrogen peroxide contents were inhibited, indicating less salt-stress toxicity. Additionally, ascorbate peroxidase, peroxidase, and superoxide dismutase activities were promoted as a reason for the single inoculation and co-inoculation with the mentioned isolates, thus boosting the tolerance of plants to cope with salinity stress. Moreover, mineral contents (except Na^+^) were enhanced in salinity-stressed canola plants in response to inoculation with *Azotobacter chroococcum*, *Alcaligenes faecalis,* and their co-inoculation. We suggest using the co-inoculation with *Azotobacter chroococcum* and *Alcaligenes faecalis* producing IAA, solubilizing phosphate, and containing ACC-deaminase as an effective and important approach for ameliorating salinity stress.

## Figures and Tables

**Table 1:** Effect of bacterial inoculation on the microbial characteristics of the rhizosphere. TBC: total bacterial count, NFC: nitrogen fixer count.

Treatment	TBC * × 10^5^ CFU/gm Dry Soil	TBC Increasing %	NFC × 10^3^ CFU/gm Dry Soil	NFC Increasing %	Dehydrogenase (μg TPF/g Dry Soil/24 h)	Dehydrogenase Increasing %
Saline soil control	78	34.4	2.3	109	112	26.9
*A. chroococcum*	96	65.5	3.6	227	144	63.3
*A. faecalis*	122	110.3	3.9	254	191	116.6
*A. chroococcum + A. faecalis*	124	113.7	3.9	254	187	112

**Table 2:** Organic solutes contents (mg g^−1^ DW) in canola plant inoculated with *Azotobacter chroococcum, Alcaligenes faecalis,* and their co-inoculation under salinity-stress conditions. Bars show means of three independent replications (n = 3) ± standard error. Means with the same letter are not significantly different at *p* < 0.05. DW: dry weight.

Treatments	Soluble Sugars	Soluble Proteins	Proline
Saline soil control	116.7 ± 3.85 b	16.9 ± 0.26 b	0.64 ± 0.04 a
*A. chroococcum*	135.3 ± 11.85 b	24.6 ± 0.48 a	0.62 ± 0.009 ab
*A. faecalis*	157.1 ± 9.76 b	25.5 ± 0.70 a	0.59 ± 0.006 bc
*A. chroococcum* + *A. faecalis*	226.5 ± 6.69 a	26.2 ± 0.38 a	0.57 ± 0.023 c

**Table 3:** Minerals’ contents (mg g^−1^ DW) in canola plant inoculated with *Azotobacter chroococcum, Alcaligenes faecalis,* and their co-inoculation under salinity-stress conditions. Bars show means of three independent replications (n = 3) ± standard error. Means with the same letter are not significantly different at *p* < 0.05. DW: dry weight.

Treatments	Na	K	N	Ca	Mg
Saline soil control	14.81 ± 0.96 a	2.34 ± 0.10 d	1.34 ± 0.06 d	0.22 ± 0.05 b	0.39 ± 0.04 b
*A. chroococcum*	7.36 ± 0.35 c	3.56 ± 0.06 c	2.12 ± 0.09 b	0.25 ± 0.04 b	0.31 ± 0.03 b
*A. faecalis*	9.24 ± 0.70 b	4.17 ± 0.08 b	1.97 ± 0.07 c	0.30 ± 0.04 b	0.71 ± 0.05 a
*A. chroococcum + A. faecalis*	6.37 ± 0.11 d	5.66 ± 0.09 a	2.41 ± 0.05 a	0.62 ± 0.08 a	0.61 ± 0.02 a

## Data Availability

No new data were created or analyzed in this study. Data sharing is not applicable to this article.
